# Interplay among Anxiety, Digital Environmental Exposure, and Cognitive Control: Implications of Natural Settings

**DOI:** 10.3390/bs14040323

**Published:** 2024-04-13

**Authors:** Viola Benedetti, Fiorenza Giganti, Maria Cotugno, Chiara Noferini, Gioele Gavazzi, Giorgio Gronchi, Stefania Righi, Francesco Meneguzzo, Francesco Riccardo Becheri, Qing Li, Maria Pia Viggiano

**Affiliations:** 1 Department of Neuroscience, Psychology, Drug Research and Child’s Health (NEUROFARBA), University of Florence, 50135 Florence, Italy; viola.benedetti@unifi.it (V.B.); fiorenza.giganti@unifi.it (F.G.); maria.cotugno1@edu.unifi.it (M.C.); noferini@lens.unifi.it (C.N.); gioele.gavazzi@unifi.it (G.G.); giorgio.gronchi@unifi.it (G.G.); stefania.righi@unifi.it (S.R.); 2 European Laboratory for Non-Linear Spectroscopy, University of Florence, Sesto Fiorentino, 50019 Florence, Italy; 3 Institute of Bioeconomy, National Research Council, 10 Via Madonna del Piano, Sesto Fiorentino, 50019 Florence, Italy; francesco.meneguzzo@cnr.it; 4 Central Scientific Committee, Italian Alpine Club, 19 Via E. Petrella, 20124 Milano, Italy; 5 Pian dei Termini Forest Therapy Station, 51028 San Marcello Piteglio, Italy; ricerca@terapiaforestale.it; 6 Department of Rehabilitation and Physical Medicine, Graduate School of Medicine—Nippon Medical School, 1-1-5 Sendagi, Bunkyo-ku, Tokyo 113-8603, Japan; qing-li@nms.ac.jp

**Keywords:** anxiety, cognitive control, response inhibition, forest therapy, Go/No-Go, natural exposition

## Abstract

Inhibitory control performance may differ greatly as a function of individual differences such as anxiety. Nonetheless, how cognitive control proficiency might be influenced by exposure to various environments and how anxiety traits might impact these effects remain unexplored. A cohort of thirty healthy volunteers participated in the study. Participants performed a Go/No-Go task before exposure to a ‘forest’ and ‘urban’ virtual environment, in a counterbalanced design, before repeating the GNG task. The State-Trait Anxiety Inventory (STAI) was finally filled-in. Our findings unveiled an initial negative correlation between anxiety trait levels and GNG task performance, consistent with the established literature attributing difficulties in inhibitory functionality to anxiety. Additionally, different environmental exposures reported opposite trends. Exposure to the ‘forest’ environment distinctly improved the GNG performance in relation to anxiety traits, while the ‘urban’ setting demonstrated adverse effects on task performance. These results underscore the intricate relationship among cognitive control, environmental exposure, and trait anxiety. In particular, our findings highlight the potential of natural settings, such as forests, to mitigate the impact of anxiety on inhibition. This might have implications for interventions aimed at improving cognitive control.

## 1. Introduction

There is an ever-growing interest in the effect that exposure to greenery has on psychophysical well-being (see for example [1]). Indeed, it is well documented that being in outdoor environments with green areas has positive effects on both mental and physical health [2,3,4]. The beneficial effects of exposure to greenery have been widely investigated in the so-called shinrin-yoku (forest bathing [5], for a review see [6]) and there is an extensive body of literature that has reported important benefits to both healthy individuals and those with clinical conditions such as affective and psychotic disorders [7] or chronic stroke patients [8]. Forest exposure has been proven to be effective in reducing stress [9,10] and cortisol levels [10,11,12], decreasing anxiety [13] and depression [14,15], regulating mood [16,17,18], and improving general health [19]. Moreover, forest exposure has shown positive effects on physiological aspects, benefiting the immune, neuroendocrine, and cardiovascular systems [20].

Another interesting aspect of research based on green space exposure is that many positive effects have also been achieved, in addition to real exposure, through virtual immersion in green environments [21]. This approach is particularly useful for individuals with limited access to nature, such as those living in urban areas with impaired mobility due to chronic disease or frailty. Virtual nature experiences have been shown to have positive effects on psychological and physiological outcomes, using various degrees of immersivity, from virtual reality (VR) protocols to media contents such as videos or images [22,23,24,25,26]. For instance, virtual exposure to forest environments reduced perceived anxiety [13]. Similarly, other studies found psychological benefits from virtual nature exposure [27]. It has been shown that the psychological effects of exposure to greenery depend on the percentage of green present in the videos: high green levels show more significant effects [28]. It must be said that the results from studies do not always converge [29,30,31,32]. This could depend on methodological differences or individual factors like age, gender, personality traits, and sleep patterns. For example, O’Meara and colleagues [33], investigating virtual nature exposure among university students with low and high anxiety, reported a significant decrease in negative affect only among high-anxiety participants.

Nature’s positive influence can be dissected to analyze its impact on specific dimensions of cognitive functions. This positive effect of green exposure on cognition has been investigated in a fine-grained manner over the last decades, reporting positive outcomes mainly on working memory, cognitive flexibility, and attentional control [21,34]. There is ample evidence to support this assertion. For instance, Pasanen and colleagues [35] found that walking in a coniferous forest improved sustained attention, and Berman and colleagues [36] reported improved directed attention abilities after walking in nature. Positive effects of greenspace exposure on attention, memory, and general intellectual functioning have also been observed in children and adolescents [37,38].

It is worth noticing that although research has focused on certain cognitive functions, more complex processes, such as inhibitory control, have received little attention. However, this executive function plays a critical role in adapting our behavior to internal and external demands of various environmental surroundings. Inhibition therefore allows us to act appropriately facing changing conditions and withholding impulses or inappropriate responses [39]. The inhibitory network and its structural and functional organization can be modulated by three factors that impact the function [40,41]. A dysregulation of this process is linked to dysfunctional behaviors observed in various psychological, neurological, and psychiatric conditions, highlighting its vital relevance. This link has been extensively explored in studies by Barkley [42] and Aron and colleagues [43], and comprehensively reviewed by Feil’s group [44]. Another clear example of maladaptive plasticity that in the long run determines a worsened baseline inhibition functioning is the case of individual differences such as trait anxiety [45,46,47,48,49,50,51,52,53]. The constant high arousal associated with trait anxiety has clear detrimental consequences [54,55,56,57,58]. Among those, high trait anxiety determines a lower efficiency of the inhibitory network default state. This is corroborated by several pieces of evidence both at the behavioral level [45,46,47,48,49,50,51,52,53] as well as by neural structural and functional evidence within the inhibitory network. Indeed, inhibitory deficiencies are reported in different inhibitory paradigms and are matched by trait-related volume alteration in several regions including the limbic and frontal areas [59,60]. At the same time, functional differences characterize crucial structures of the saliency network, such as the insula and the anterior cingulate cortex (ACC) or the dorsolateral section of the prefrontal cortex [61,62,63,64]. The typical worry state of trait anxiety therefore leads to a frailty of the inhibitory system, running on higher demands moved by the hyper relevance of external monitoring for threat and by the necessity of compensatory frontal resources. Although a beneficial effect of natural exposure on inhibition has been documented [65], whether—and to what extent—the effectiveness of different environmental influences may vary as function of inhibition proficiency at baseline remains of particular interest given the variability across individuals.

In this study, the network baseline was accounted for using as a proxy trait anxiety, which could potentially mediate the magnitude of the observed effect. Considering that trait anxiety partially correlates with states of anxiety [66], we have included its measurement to ensure that our data align with the existing literature. The presence of a correlation between trait and state anxiety would not only confirm the consistency of our findings with established research but also enable us to assess the potential presence of confounding factors on environmental exposure effects. In fact, a lack of correlation between measures would indicate potential discrepancies, e.g., as in individuals with low trait anxiety experiencing momentarily high levels of state anxiety.

Therefore, here we explored—through virtual exposure to natural and urban environments—inhibitory control via a Go/No-Go Task while assessing anxiety via the State-Trait Anxiety Inventory (STAI).

## 2. Materials and Methods

### 2.1. Participants

We enrolled a total of thirty healthy volunteers (18 men; mean age = 23.60 ± 2.79 standard deviation (S.D.); range = [21–33 years old]) with no history of neurological and psychiatric illness or drug abuse, with normal hearing and normal or corrected-to-normal vision. Additionally, they were instructed to refrain from consuming caffeine for at least three hours before the experiment. Testing was performed over a period spanning from the 30 January 2023 to the 28 March 2023. Data from this sample have already been published in our previous work [65]. This study was performed according to the Declaration of Helsinki and was approved by the Ethical Committee of the University of Florence (No. 253, 2023). Before the experiment, each subject was blind to the purpose of this study, which was carefully explained afterward. The enrolling of participants was conducted in conformity with the approved ethical committee by the diffusion of flyers in public locations. To obtain a reliable sample size, an a priori power analysis was conducted based on the two-tailed correlation test with the software G*Power 3.1.9.7. Considering an a priori effect size of 0.50, α = 0.05, we measured a power of 0.86 for a sample size of 30 subjects.

### 2.2. Materials

#### 2.2.1. Video

Two videos were administered, one featuring forest scenery and the other urban scenery. The video length was five minutes each and the video resolution corresponded to 3686 × 2304 pixels. Both videos reported scenes without human presence. All scenes were filmed with a stationary camera, using panoramic shots.

The ‘forest’ condition video represented forest landscapes situated in the Apennine Mountains (43°57′ N, 11°10′ E and 44°01′ N, 11°00′ E). The footage included coniferous beech trees and water streams. In particular, five scenery shots were subsequently presented throughout the video, one-minute length each. Gatherings of trees were depicted in each shoot without the interference of external elements (i.e., animals or anything else). Movement in the scene was solely driven by the wind and the resulting movement of leaves in the environment. Out of five scenes, two featured trees with green vegetation, while the others depicted autumnal scenes with bare trees and fallen leaves on the ground. In one of these scenes, a stream was present. All scenes were shot perpendicular to the ground, capturing the trunks of the trees laterally and their canopies. One of the scenes showed a low-angle panoramic view exclusively of the tree canopies. For each scene, the corresponding sounds recorded in the environment are included. These included sounds of the wind flowing through the leaves and bird calls; in the scene with the stream, the sound of water flowing was included.

The ‘urban’ condition video consisted of urban environments. Urban sceneries were recorded in downtown Prato (Italy). The footage included building scenes, such as offices, front doors, and windows. In detail, the urban video also comprised five consecutive scenes, each lasting approximately one minute. Each scene featured a city architectural structure; specifically, three out of the five scenes depicted close-up views of urban elements, such as a house door, first-floor office windows, and the roof of a building. The remaining scenes captured buildings from a more distant perspective, encompassing the entire facades of the structures. The scenes were shot perpendicular to the ground or using low angle framing to capture the height of the buildings. There were no moving elements in the videos (e.g., no vehicles). Shoots included respective audio, encompassing industrial and car sounds.

#### 2.2.2. Task

**Figure 1:**
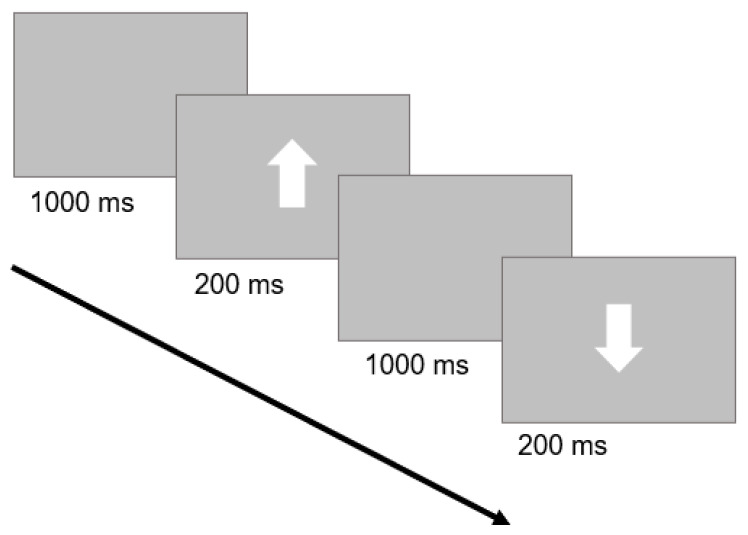
Trial structure of the GNG task: a ‘Go Stimulus’ trial, followed by a ‘No-Go Stimulus’ trial, is depicted. A blank screen was followed by a white arrow. Subjects were instructed to tap quickly and accurately for upward arrows (‘Go Stimulus’) and to withhold their response for downward arrows (‘No-Go Stimulus’).

The GNG task assesses our inhibitory control ability by measuring our capacity to withhold inappropriate responses. This task was initially validated by the seminal studies of Donders (see [67]) and has been extensively replicated over time. We applied a version similar to other works published by our group [65,68,69]. Briefly, the visual targets were white arrows presented on a gray background at the center of a touch-screen monitor. Arrows could either point up or down with respect to the monitor’s horizontal axis. Upward arrows corresponded to the ‘Go Stimulus’ and participants were instructed to tap directly on the arrow as quickly and accurately as possible. Conversely, downward arrows corresponded to the ‘No-Go Stimulus’ and subjects were instructed to suppress the response. Each arrow remained on screen for 200 ms. A blank screen was presented for 1000 ms between arrows (Figure 1). The task consisted of 100 trials in total (80 ‘Go Stimulus’ trials and 20 ‘No-Go Stimulus’ trials). The presentation order of trials was randomized. The paradigm was preceded by a short training phase of 8 trials (6 ‘Go Stimulus’ trials, 2 ‘No-Go Stimulus’ trials). The task was coded with custom python code on OpenSesame 3.2.6 Kafkaesque Koffka [70].

#### 2.2.3. Questionnaire

Trait and state anxiety were quantified by means of the Italian version of the State-Trait Anxiety Inventory (STAI), with, respectively, the X2 and X1 scales [71,72]. Each scale had items rated on a 4-point Likert scale. The scores ranged between 20 and 80.

#### 2.2.4. Procedure

**Figure 2:**
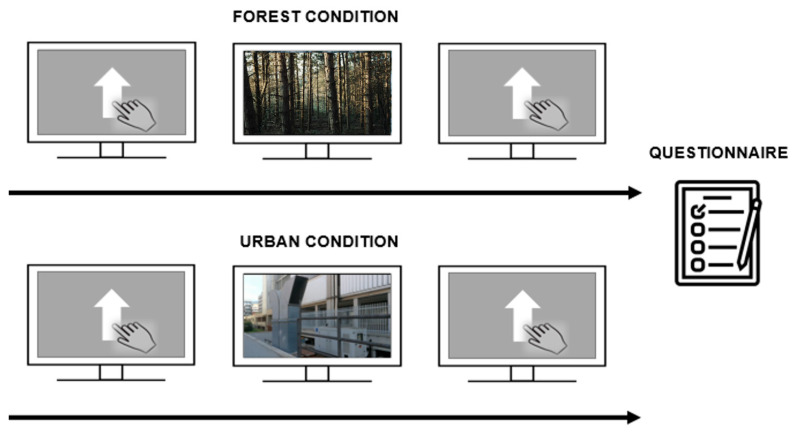
Experimental procedure. The ‘forest’ and ‘urban’ conditions are depicted on the upper and lower parts of the panel, respectively. The experimental procedure corresponded between sessions, except for the video presented. The GNG was performed first (pre-video), the video was then administered, and then the task was administered again (post-video). At the end of the last session, participants were asked to fill in the STAI questionnaire.

The study consisted of two sessions that were executed one week apart. Subjects were positioned 57 cm away from the monitor. Sessions started with participants initially performing a GNG task. Subsequently, the video was administered and subjects were asked to carefully watch and pay attention to the video of the ‘forest’ or ‘urban’ condition. After watching the video, participants once again performed the GNG task. Finally, at the end of the last session, participants were asked to fill in the STAI questionnaire (Figure 2). Video contents (i.e., ‘forest’ and ‘urban’ conditions) between sessions were counterbalanced between participants. More precisely, half of the sample was initially exposed to the forest environment and then to the urban one, whereas the rest of the participants followed the opposite order of administration.

### 2.3. Analysis

For each subject, the STAI-X1 (the scale of state anxiety) and STAI-X2 (the scale of anxiety trait) were scored. To control for potential discrepancies between the scales, a bootstrap Pearson correlation was performed. For the scale X2, we additionally measured an Alpha Cronbach = 0.47. In the GNG task, behavioral performance was first quantified by the following measures: Go mean reaction times (‘Go RTs’), percentage of correct responses in the Go condition (‘Go accuracy’), and the number of inhibitory failures in the No-Go condition (‘No-Go commission errors’). Measures were quantified for the pre- and post-video GNG runs. For each measure, we selected the value obtained at the pre-video run, in the first session, that the subject performed. These values were considered the ‘baseline’ condition, as they reflect the reference performance while being *naïve* to task rules and experimental procedure. The Δ of each measure (i.e., post *minus* pre-video) was then calculated in both conditions (i.e., ‘forest’ and ‘urban’). Additionally, before hypothesis testing, outliers above or below 3 S.D. from the mean were eliminated from each measure. Firstly, for the ‘baseline’ condition, we performed a bootstrap Pearson correlation with STAI-X2 scores and each GNG measure (i.e., ‘Go RTs’, ‘Go accuracy’ and ‘No-Go commission errors’). Subsequently, for both ‘forest’ and ‘urban’ conditions, a bootstrap Pearson correlation with the STAI-X2 scores and the Δ of each measure was performed. Bootstrap is a robust statistical method that allows for the creation of confidence intervals (C.I.) for correlations [73]. Each bootstrap analysis consisted of 10,000 iterations. All tests were two-tailed with a Bonferroni-corrected alpha level set at *p* ≤ 0.025. Statistical hypothesis testing was performed using R (version 4.3.1; R Core Team 2023 [74]) using the ‘bootcorci’ package [73].

## 3. Results

The STAI-X2 (trait anxiety) mean score corresponded to 45 ± 9.5 S.D., with scores ranging from 27 to 62. The STAI-X1 (state anxiety) mean score was 36 ± 7.2 S.D., in a range from 22 to 50. As expected, the two scales reported a significant positive correlation with an estimate of 0.51 (C.I. = [0.17; 0.75]; *p* < 0.001). Descriptives of the behavioral performance in the GNG task are reported in Table 1.

**Figure 3:**
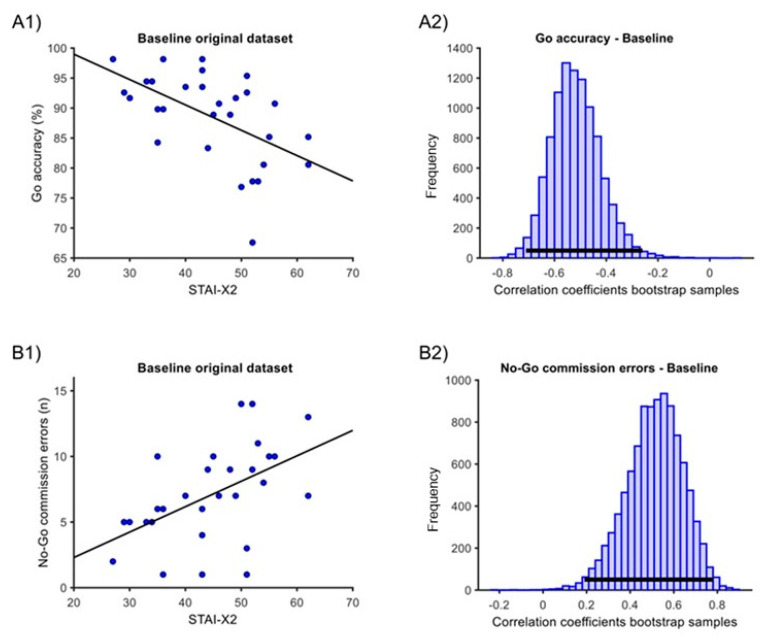
Correlation between STAI-X2 scores and GNG measures in the ‘baseline’ condition. (**A1**) Original dataset correlation of STAI-X2 scores with ‘Go accuracy’. (**A2**) Associated Bootstrap sample distribution of Pearson correlation coefficients. (**B1**) Original dataset correlation of STAI-X2 scores with ‘No-Go commission errors’. (**B2**) Associated Bootstrap sample distribution of Pearson correlation coefficients. In (**A1**,**B1**), the dark line corresponds to linear trends. In (**A2**,**B2**), the horizontal dark line indicates the 95% C.I.

In the ‘baseline’ condition, one outlier was eliminated for the ‘Go RTs’ measure. At baseline, we found significant correlations of STAI-X2 with both ‘Go accuracy’ and ‘No-Go commission errors’ measures. For ‘Go accuracy’, there was a negative sample correlation of −0.52, with a 95% percentile bootstrap C.I. of [−0.71; −0.26] and a *p* < 0.001 (Figure 3(A1,A2)). For the ‘No-Go commission errors’, there was a positive sample correlation of 0.47, with a C.I. of [0.15; 0.74] and a *p* = 0.001 (Figure 3(B1,B2)). The correlation with ‘Go RTs’ was not significant (estimate = −0.12, C.I. [−0.52; 0.28], *p* = 0.478).

**Figure 4:**
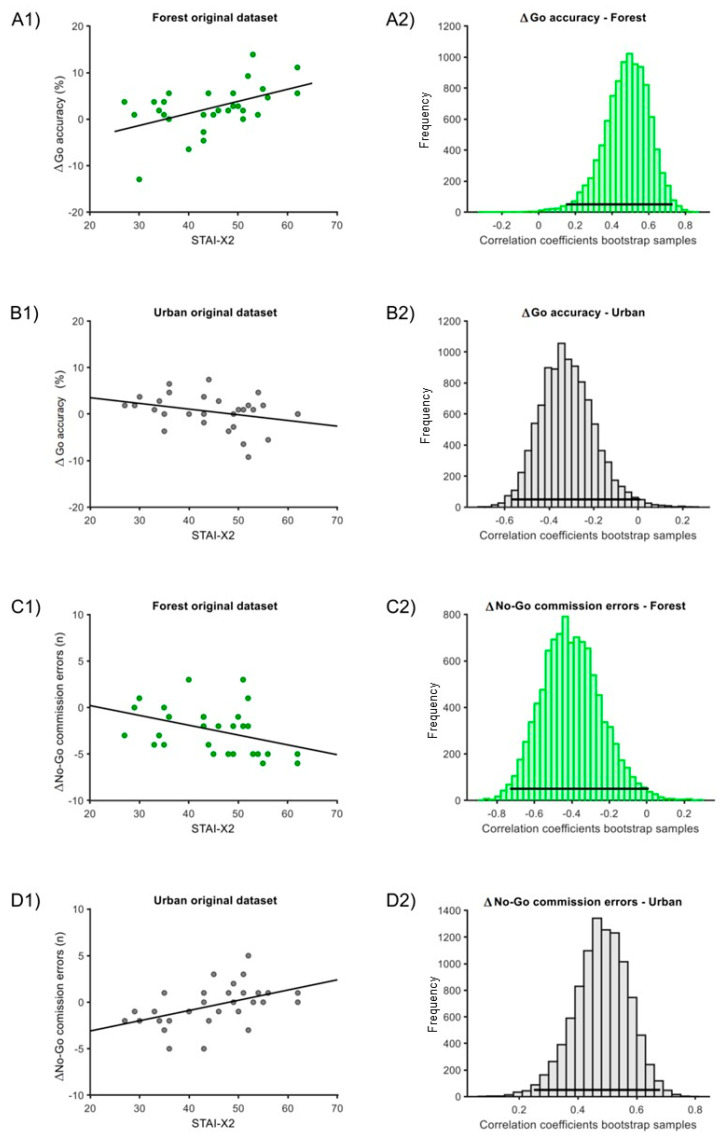
Correlation of STAI-X2 scores and Δmeasures for the ‘forest’ and ‘urban’ conditions. (**A1**) Original dataset correlation of STAI-X2 scores with ΔGo-accuracy for the ‘forest’ condition. (**A2**) Associated bootstrap sample distribution of Pearson correlation coefficients. Panel (**B1**) Original dataset correlation of STAI-X2 with ΔGo-accuracy for the ‘urban’ condition. Panel (**B2**) Associated bootstrap sample distribution of Pearson correlation coefficients. (**C1**) Original dataset correlation of STAI-X2 scores with Δ No-Go commission error for the ‘forest’ condition. (**C2**) Associated bootstrap sample distribution of Pearson correlation coefficient. (**D1**) Original dataset correlation of STAI-X2 scores with ΔNo-Go commission error for the ‘urban’ condition. (**D2**) Associated bootstrap sample distribution of Pearson correlation coefficients. In (**A1**,**B1**,**C1**,**D1**), the dark line corresponds to linear trends. In (**A2**,**B2**,**C2**,**D2**), the horizontal line indicates the 95% C.I.

For the Δ correlation analysis, in the ‘forest’ condition, one outlier was eliminated for the ‘ΔGo accuracy’ measure. In the ‘urban’ condition, one outlier was eliminated for the ‘ΔGo accuracy’ and one for the ‘ΔGo RTs’ measures. In both the ‘forest’ and ‘urban’ conditions, we found significant correlations of STAI-X2 with either ‘ΔGo accuracy’ and ‘Δno-Go commission errors’. Particularly, the correlation with ‘ΔGo accuracy’ for the ‘forest’ condition was positive (estimate = 0.49, C.I. = [0.15; 0.73], *p* = 0.002; Figure 4(A1,A2)), while for the ‘urban’ condition, the correlation was negative (estimate = −0.32, C.I. = [−0.57; 0.01], *p* = 0.019; Figure 4(B1,B2)). Moreover, the correlation with ‘ΔNo-Go commission errors’ for the ‘forest’ condition was negative (estimate = −0.40, C.I. = [−0.73; 0.01], *p* = 0.024; Figure 4(C1,C2)), while it was positive for the ‘urban’ condition (estimate = 0.47, C.I. = [0.24; 0.68], *p* < 0.001; Figure 4(D1,D2)). The correlation with ‘ΔGo RTs’ did not report any significant differences (‘forest’ condition: estimate = 0.02, C.I. = [−0.34; 0.37], *p* = 0.86; ‘urban’ condition: estimate = 0.22, C.I. = [−0.57; 0.18], *p* = 0.20).

## 4. Discussion

In this study, we explored for the first time whether exposure to different environments may influence cognitive control differently, depending on the individual’s trait anxiety.

Initially, we examined data at baseline (before each environmental exposure), observing a negative correlation between anxiety levels and participants’ performance on the Go/No-Go (GNG) task. This finding is in line with the literature [45,46,47,48,49,50,51,52,53] as it suggests that individual differences such as anxiety traits lead to worsening cognitive control defaults.

In fact, according to the Attentional Control Theory [75], inhibition efficiency might be impaired due to a disrupted balance between the goal-directed and externally stimulus-driven cognitive systems, fostering this latter one. Trait anxiety is indeed linked to a heightened continuous arousal state, diverting attention to eventual external threats, depleting our attentional resources. This would divert our cognitive focus towards internal or external stressors, rather than the task to be performed. In the long run, trait anxiety determines structural and functional changes in the inhibitory network [59,60,61,62,63,64], determining overall a decreased default behavioral efficiency [45,46,47,48,49,50,51,52,53] that seems observable in our baseline data.

Notably, the effects’ magnitude of different environmental exposures on GNG performance differs as function of anxiety traits. We found that exposure to different virtual environments, such as a forest or urban virtual settings, influences the accuracy of the GNG task performance. In particular, the forest environment improved participants’ “Go accuracy” and reduced “No-Go commission errors” to a greater extent in participants with a high anxiety trait. At variance, urban exposure appeared to induce detrimental consequences in highly anxious participants’ performances.

Our results highlight how anxiety traits modulate the impact of different environments in cognitive control, underscoring the significant role that natural settings, like forests, may play in mitigating the effect of anxiety on this function. The positive impact of natural environments, such as forests, on anxiety and psychophysiological well-being is well documented [13,76,77,78]. It is noteworthy—and consistent with previous results in other cognitive domains such as attention [22,79,80]—how these natural settings appear to counteract factors that lead to poor performances in anxious individuals. One potential explanation may reside in the reduction in cognitive fatigue exerted by the natural environments. This possibility is in line with the Attention Restoration Theory (ART), which suggests that natural scenery would induce a state of ‘soft fascination’ with fewer demands to be processed in terms of directed attention, allowing our cognitive resources to be replenished [81,82]. We can speculate that the previously observed positive effects of nature on inhibitory control [65] are further enhanced in anxious individuals possibly due to resource restoration, which is especially crucial in anxiety where our resources are depleted. In fact, the inhibitory system of anxious individuals’ results depleted to a great extent due to constant system imbalance. Additionally, the negative effects of urban exposure on anxious participants support this interpretation, as urban environments are often perceived as overwhelming and associated with high attentional demands, possibly exacerbating the cognitive strain in anxious individuals.

These results point out an enhanced overall vulnerability as a function of trait anxiety. In this case, it seems that an initial inhibitory frailty determines an enhanced permeability of the network to environmental modulations, both in positive and negative directions.

This interplay among nature, anxiety, and cognitive control may not only have behavioral manifestations but could also be linked to specific neural mechanisms. This deserves further exploration to gain a comprehensive understanding. All the main models on inhibitory control assume two core components responsible for optimizing and adapting our behavior to the environment: an excitatory and an inhibitory component [83,84,85,86,87]. As attentional control theories support, we hypothesize that trait anxiety negatively impacts the excitatory component of the inhibitory system by over-clocking the hub due to the excessive external orientation [75]. Delving into the possible involvement of the neural underpinnings of these findings, we may hypothesize which brain regions might be responsible for these findings. Consistent with the literature and according to the most recent model of cognitive control (see [83]), trait anxiety seems to negatively impact the excitatory component of our inhibitory system, possibly due to the increased attention to the external environments. Trait anxiety might hyper-activate the limbic system (e.g., amygdala, insula, and other structures involved in saliency and vigilance processing) and thalamus [88,89]. These brain regions are extensively reported as belonging to the excitatory component of cognitive control [83]. The hyper-activation of the previously mentioned areas would determine an imbalance in our inhibitory/excitatory system relationship, leading to the observed inhibitory failures at baseline (before any environmental exposition). However, we have also observed how exposure to the forest environment might help in restoring this balance by possibly reducing the excessive activity in the excitatory hub and/or promoting top-down control by frontal areas, such as the inferior/middle or superior frontal gyri. In such a frame, we might think that natural environments could normalize the inhibitory system activity and thus improve cognitive control in individuals where inhibition is more challenging and onerous, as in anxiety traits. However, this is just a speculation requiring more research on neural correlates to be confirmed. Additionally, in future studies, there will be a need to control the different mediums of exposure and content specificity. Since in the present study we have the limitation of not having compared the physical exposition to the digital one, one possibility would be to examine the effects of different environments (physical vs. digital) and different exposures to media content. This would allow us to explore the impact of various relaxing environments and content specificity beyond just the forest, by taking into account the possible contribution of attention. Furthermore, it is important to include a neutral content as a baseline among the various video contents being tested. This would help to control for confounding variables that may influence the outcome of the experiment. At last, further research should address whether different context exposures may affect the relationship between cognitive control and momentary oscillations in anxiety.

In conclusion, this study explored the intricate relationship between inhibitory individual differences, indexed by trait anxiety, and the impact of exposure to natural versus urban virtual environments. At baseline, we confirmed the negative correlation between anxiety trait levels and performance on the Go/No-Go (GNG) task, in line with existing research that suggests anxiety’s potential to disrupt cognitive control [45,46,47,48,49,50,51,52,53]. Additionally, our findings revealed a crucial nuance in this relationship. The association between anxiety and GNG performance is not consistent across different environmental exposures. Exposure to natural settings, particularly a forest environment, had a positive impact on highly anxious participants. Importantly, these findings might have important practical implications, as they could inform specific interventions and preventive measures aimed at addressing cognitive performance issues related to conditions such as anxiety, especially in cases with problems acceding to the forest environment for locomotor issues, architectonical barriers, or specific mental conditions (e.g., agoraphobia).

By understanding the dynamic interplay between cognitive control proficiency, anxiety, and the environment, it would be possible to develop strategies that harness the potential of natural settings to improve cognitive function in individuals dealing with anxiety traits.

## Figures and Tables

**Table 1:** GNG measures (means ± S.D.).

Measures	Baseline		
Go RT (ms)	447.3 ± 57		
Go accuracy (%)	88.9 ± 8		
No-Go commission (n)	6.9 ± 4		
	**Pre-video**	**Post-video**	**Δ**
	**Forest**		
Go RT (ms)	427.0 ± 64	434.5 ± 95	7.5 ± 80
Go accuracy (%)	88.5 ± 11	90.2 ± 12	1.7 ± 6
No-Go commission (n)	6.6 ± 4	4.2 ± 3	−2.4 ± 3
	**Urban**		
Go RT (ms)	435.4 ± 73	420.0 ± 60	−15.4 ± 43
Go accuracy (%)	89.8 ± 11	89.8 ± 11	0.0 ± 5
No-Go commission (n)	5.2 ± 3	4.8 ± 4	−0.4 ± 2

## Data Availability

Data are available from the corresponding author upon request.
